# AKUDENTAL teeth instance segmentation dataset: a cross-dataset analysis

**DOI:** 10.1186/s12903-025-07645-0

**Published:** 2026-01-12

**Authors:** Melih Oz, Aycan Sengul, Mukerrem Hatipoglu, Taner Danisman

**Affiliations:** 1 Computer Engineering Department, Faculty of Engineering, Akdeniz University, Dumlupınar Blvd, Antalya, 07070 Türkiye; 2 Medical Imaging Program, Vocational School of Health Services, Akdeniz University, Dumlupınar Blvd, Antalya, 07070 Türkiye; 3 Department of Periodontology, Faculty of Dentistry, Akdeniz University, Dumlupınar Blvd, Antalya, 07070 Türkiye

**Keywords:** Dental X-ray dataset, Cross-dataset evaluation, Artificial intelligence, Medical imaging, Instance segmentation, Object detection

## Abstract

**Background:**

Artificial Intelligence (AI) is reshaping diagnostics and disease prevention in the dental domain. Panoramic X-ray imaging is central to this progress but demands large, high-quality annotated datasets. We therefore present AKUDENTAL, a new dataset for instance segmentation of dental radiographs, to serve as a resource for model development and to assess the challenges of generalizability.

**Methods:**

We annotated 333 panoramic images, labeling 9,956 structures across 32 individual teeth and three restorative categories: implants, bridges, and crown–filling. We established semantic segmentation, object detection, and instance-segmentation baselines using UNet, DeepLabV3 + , YOLOv11, and Mask R-CNN models. Generalizability was assessed via 5-fold cross-validation and a cross-dataset evaluation on the Tufts, DENTEX, and Dual-labeled datasets.

**Results:**

A cross-dataset evaluation on the Tufts, DENTEX, and Dual-labeled datasets revealed that variations in annotation protocols are a significant factor contributing to performance differences. The cross-dataset evaluation demonstrated widely varying performance, with mean Average Precision (mAP) scores for multiclass detection ranging from a low of 0.34 on the DENTEX dataset to 0.71 on the Dual-labeled dataset Our analysis illustrates how such discrepancies can impact the interpretation of model performance.

**Conclusions:**

The AKUDENTAL dataset provides a robust new resource for the field. The performance disparities revealed in our cross-dataset analysis are not model limitations but instead strengthen the argument that annotation inconsistencies are a critical barrier to developing universally applicable AI. This highlights the imperative for broader standardization in data annotation, extending beyond tooth identification to encompass common dental procedures and restorations.

## Introduction

Advances in dental diagnostics increasingly depend on computed tomography (CT), periapical radiographs, bitewing radiographs, and panoramic X-rays to enhance clinical decision making and treatment planning. Dental imaging modalities are frequently analyzed manually, making them susceptible to human errors such as fatigue, misinterpretation, and interobserver variability. AI-assisted decision systems can accelerate the treatment process, enhance diagnostic accuracy, and reduce practitioners’ workload, ultimately improving patient care. These factors highlight the need for intelligent systems that can aid clinicians in diagnosing dental radiographs with greater accuracy and efficiency. The aforementioned systems require high-quality dental datasets to train [1, 2].

### Challenges and the need for high-quality dental datasets

Dental imaging datasets encounter fundamental challenges, including data inconsistencies, a lack of standardization, and restricted general availability. Multiple studies rely on privately labeled datasets, deterring direct comparisons between models. One important issue is the lack of extensive annotations in existing datasets. They frequently lack detailed labeling for teeth. Dental procedures such as implants, fillings, and bridges are not labeled. The resulting sparsity of details hinders the accurate detection and analysis of these structures. Another challenge derives from the way dental procedures are annotated. Teeth numbering and dental procedures (such as restorations or pathologies) are labeled separately in some datasets, which typically necessitates two-stage models, one for detecting teeth and another for categorizing their conditions [1, 3, 4]. Furthermore, many dental imaging datasets are small, proprietary, or lack expert annotations, making it challenging to train robust deep learning models [5]. They often display pronounced class imbalances and biases, and conditions like cavities or missing teeth are overrepresented, leading to skewed model predictions [2]. Addressing these challenges is vital for improving AI-based dental diagnostics and ensuring reliable solutions. To this end, we endorse extending the number of annotated classes beyond the current 32, encouraging the development of more informative systems.

## Related work

Multiple studies have introduced panoramic dental X-ray datasets that vary widely in size, annotation detail, and accessibility as summarized in Table 1.

**Table 1:** Comparison of publicly available panoramic dental X-ray datasets. “Instance Seg.” indicates whether the dataset distributes per-tooth instance masks (COCO polygons); “Available” refers to unrestricted academic access

Dataset	Images	Classes	Dental Procedures	Inst. Seg	Available
UFBA–UESC OdontoAI [6]	4,000 (850 ann.)	52 (Primary and perm. teeth)	Implants ignored	Yes	Yes
Tuzoff et al. (2019) [7]	1,574	32	Procedures ignored	No	No
Leite et al. (2021) [8]	153	16	Procedures ignored	No	No
Tufts [1]	1,000	52 (Primary and perm. teeth)	Implants numbered	Yes	Yes
Wang et al. (2025) [5]	4,000 (900 ann.)	2	Included with teeth	No	Yes
DENTEX [3]	634	32 + 4	Pathology labels sep	No	Yes
Beser et al. (2024) [9]	3,854	52 (Primary and perm. teeth)	Not specified	Yes	Yes
Dual-labeled [4]	5,000(2066 released)	33 (Num) + 7 (State)	Pathology labels sep	Yes	Yes
AKUDENTAL (Ours)	333	35	Fillings, crowns, bridges and implants included	Yes	Yes

Jader et al. (2018) first introduced the UFBA-UESC dataset, modifying segmentation masks for instance-level detection [10]. While demonstrating the potential of deep learning in dental instance segmentation, this dataset contained 1,500 images, of which 193 were annotated. Silva et al. (2020) expanded this dataset, incorporating tooth numbering alongside segmentation [11]. This work increased the number of annotated images to 543 and developed the class set to 32 for each tooth. Pinheiro et al. (2021) further refined segmentation techniques by integrating the PointRend module, which iteratively refines high-resolution predictions, improving boundary precision, especially for deciduous teeth, which were previously under-represented [6]. Silva et al. (2023) later introduced a new dataset by adding 2,507 images to the UFBA-UESC dataset, resulting in 4,000 images [6]. The dataset includes labels for primary and secondary teeth but excludes labels for implants, restorations, and other dental applications. Tuzoff et al. (2019) used a private dataset containing 1,574 images (1,352 for training and 222 for testing) with 32 classes [7]. Leite et al. (2021) introduced a 153-image dataset for segmentation that cropped into 3576 teeth separated into 16 classes [8]. Krois et al. (2021) incorporated a private dataset of 5,004 radiographs. In their study, teeth are represented by a single point in the middle [12]. Panetta et al. (2021) introduced Tufts, a multimodal dataset containing 1,000 panoramic radiographs labeled for instance segmentation, abnormalities, eye-tracking, and textual descriptions [1]. The dataset has annotated implants, but not as a separate class. Beser et al. (2024) created a dataset of 3,854 Panoramic X-ray images from pediatric patients in mixed dentition was assembled. Deciduous and permanent teeth were annotated. In total, 157,377 instance labels were created [9]. Hamamci et al. (2023) presented the DENTEX dataset, which comprises a total of 2,332 images; of these, a subset of 634 high-resolution panoramic radiographs is numerically labeled for object detection, containing 23,999 individual tooth instances [3].

According to a standardized protocol, a rectangular bounding box for each tooth is provided alongside one of five diagnostic labels—healthy, deep caries, restoration, root canal treatment, or impaction. Wang et al. (2025) presented a multimodal dataset for semi-supervised tooth segmentation, incorporating 4,000 images alongside 900 segmentation masks [5]. The dataset is categorized into separate subsets for children and adults, but it contains only two classes: teeth and background, with a fixed resolution of 640×320. Zhou et al. (2024) introduced a dual-labeled dataset comprising 5,000 panoramic radiographs for segmentation, numbering, and state assessment; of these, 2,066 samples were made publicly available and included in our analysis [4]. This publicly available dataset provides instance segmentation masks and assigns two labels to each tooth: a number based on the FDI notation and a state from one of seven categories: Tooth without anomalies, Tooth with fillings, Tooth with root canal treatment, Tooth with crown, Tooth with caries, Residual root, and Tooth with RCT and crown.

**Fig. 1:**
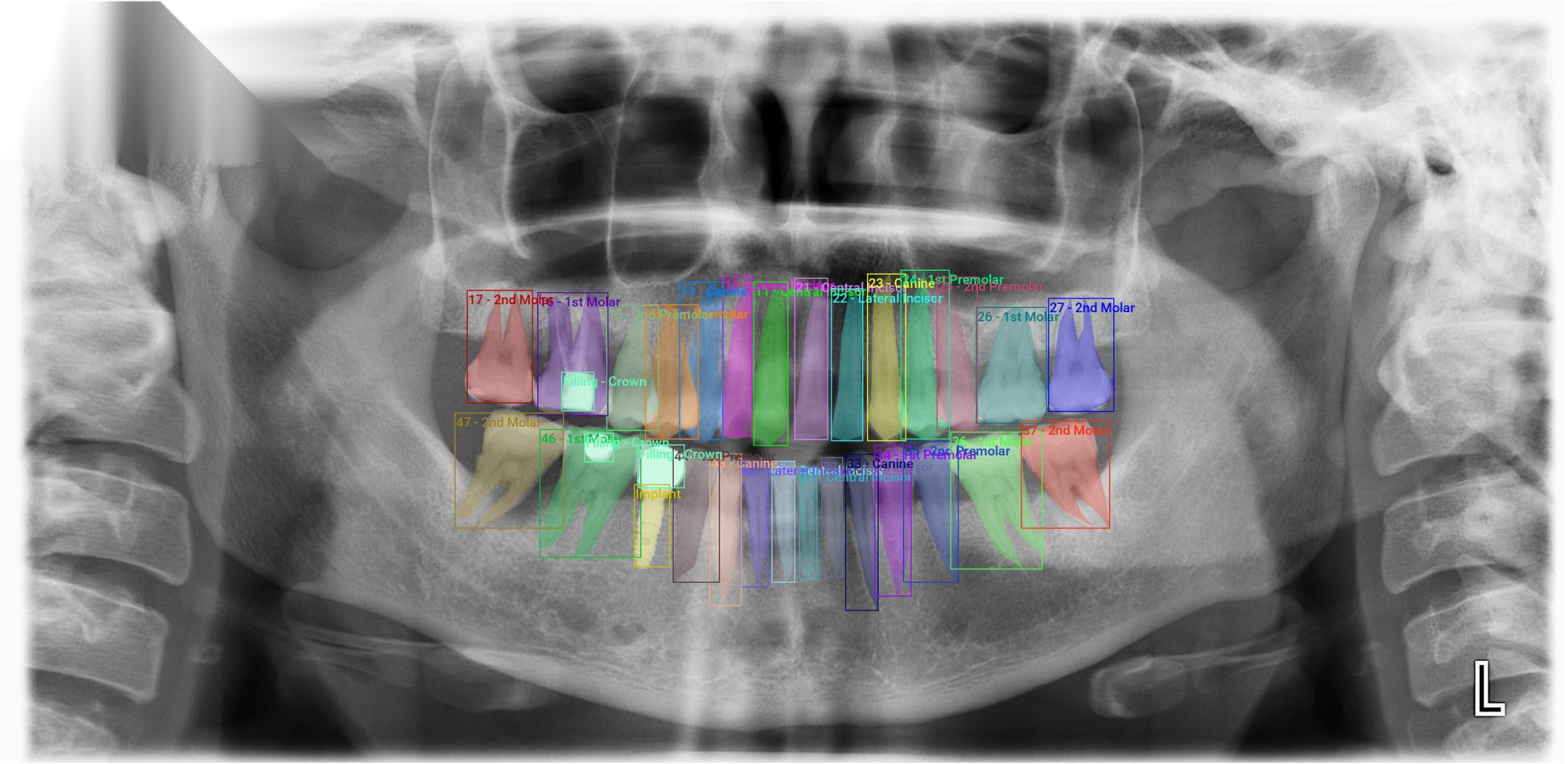
A sample from the AKUDENTAL dataset

We introduce AKUDENTAL an instance segmentation dataset containing 333 panoramic X-ray images, each annotated by an expert for individual teeth, restorations and implants. It is designed for multiclass semantic segmentation, instance segmentation, and object-detection research, offering a balanced distribution across tooth types and dental procedures. This balance delivers a reliable basis for training and evaluating models that tackle the varied tasks in dental imaging. We strive to decrease the gap between research and clinical practice, supporting the development of dependable AI diagnostic mechanisms that improve accuracy, efficiency, and accessibility in dental imaging. Additionally, our dataset enables cross-dataset benchmarking by evaluating model performance on external datasets, delivering a baseline for generalization. Fig. 1 shows an annotated X-ray sample from our dataset.

## Methodology

### AKUDENTAL dataset

The AKUDENTAL dataset comprises dental panoramic X-ray images with instance-level, polygon-based annotations that capture various tooth types and dental conditions. Initially, 700 anonymized images were collected using two panoramic X-ray devices at Akdeniz University Dental Hospital between June 20, 2022, and August 20, 2022. The data collection was conducted under ethical approval from the Akdeniz University Clinical Research Ethical Committee (Approval No: KAEK-72), and all personal identifiers were removed to ensure patient privacy.

**Fig. 2:**
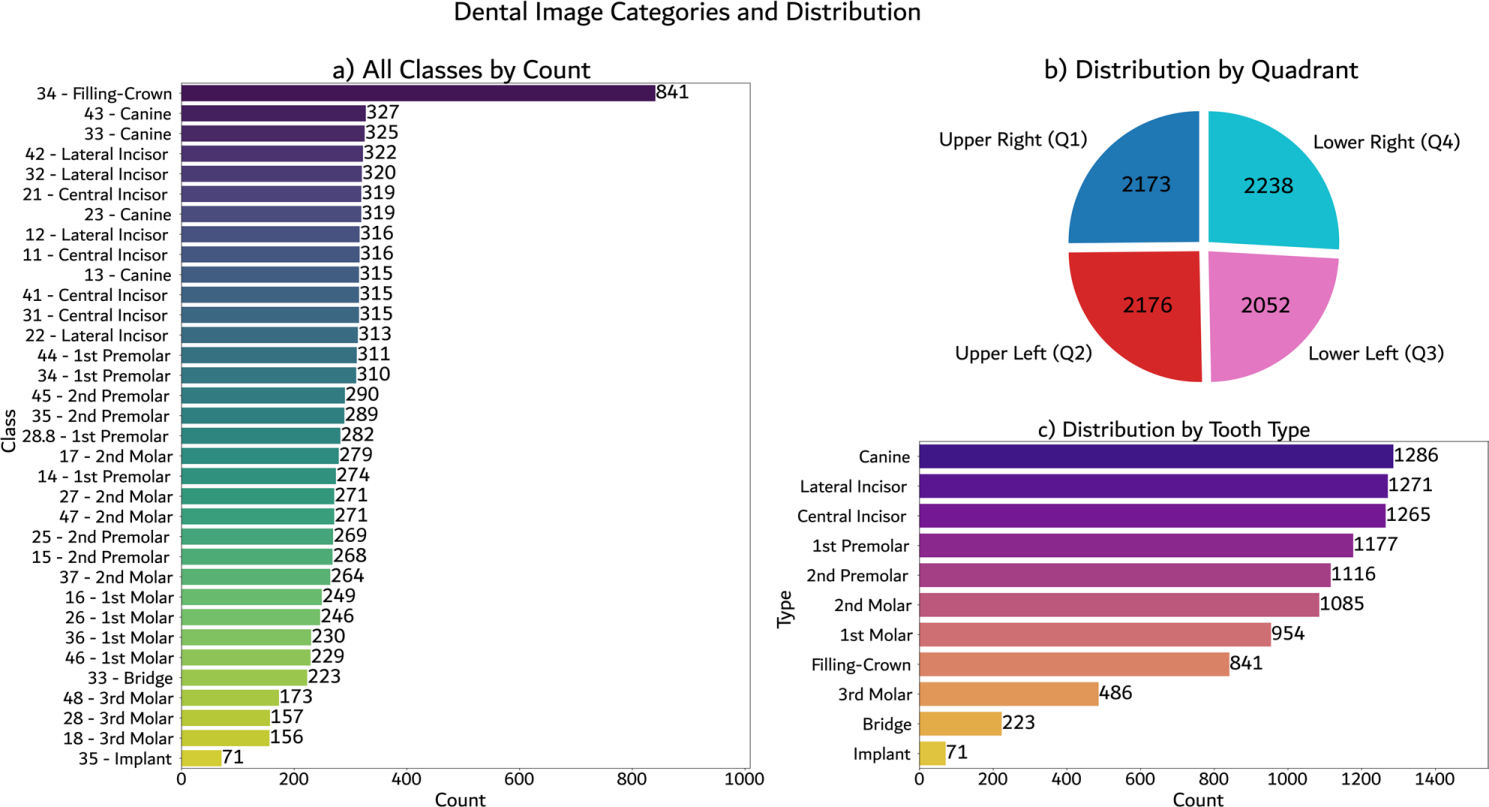
Summary statistics of the AKUDENTAL dataset: **a** Number of annotated instances per class, **b** distribution of annotations by dental quadrant, and **c** distribution by tooth type

The dataset was filtered to ensure a varying range of image representation and to minimize redundancy. Image dimensions range from 2494×1435 pixels to 2871×1536 pixels, with an average resolution of approximately 2908×1445 pixels. This high resolution supports precise, pixel-level dental segmentation. The final dataset includes 190 female and 143 male adult subjects; primary and supernumerary teeth are excluded. Fig. 2 illustrates the distribution of annotated classes, their quadrant-level breakdowns, and overall tooth type frequencies.

### Annotation protocol

**Fig. 3:**
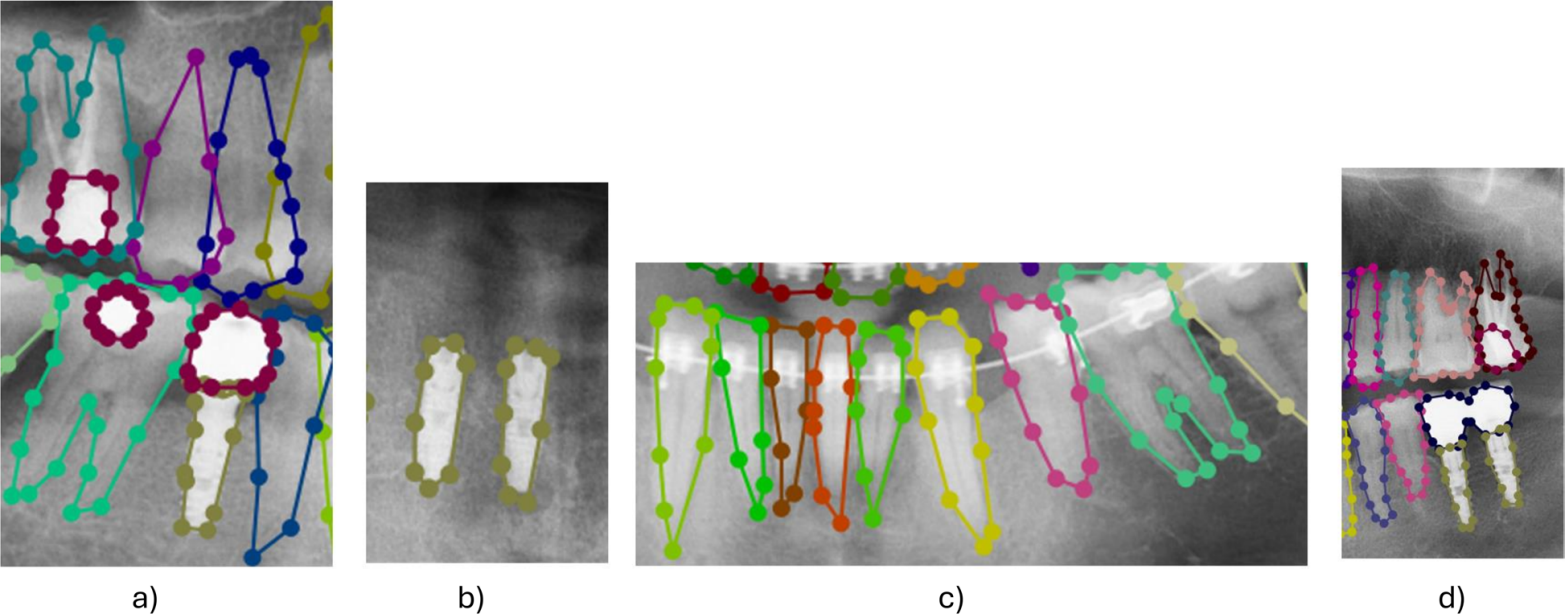
Examples of annotation scenarios **a** Implant with crown attached, **b** Implant without crown, **c** Ignored dental braces, **d** Ignored root canal treatments (also illustrating an example bridge)

Annotations were created using Labelme software with polygon-based segmentation. The annotation followed the FDI numbering conventions. Table 2 shows class-mapping and FDI number equvalent. Each dental structure was carefully outlined, including teeth, fillings, implants, and crowns. Intersections between adjacent teeth were deliberately annotated at shared boundaries, ensuring both neighbouring teeth include the common region in their annotations. Fig. 3a shows a sample crossing boundary. This conservative boundary approach supports fine-grained learning and overlap-aware model training [13].

**Table 2:** Class id mapping

Class ID	FDI Number	Tooth Name
0	11	Central Incisor
1	12	Lateral Incisor
2	13	Canine
3	14	1st Premolar
4	15	2nd Premolar
5	16	1st Molar
6	17	2nd Molar
7	18	3rd Molar
8	21	Central Incisor
9	22	Lateral Incisor
10	23	Canine
11	24	1 st Premolar
12	25	2nd Premolar
13	26	1 st Molar
14	27	2nd Molar
15	28	3rd Molar
16	31	Central Incisor
17	32	Lateral Incisor
18	33	Canine
19	34	1 st Premolar
20	35	2nd Premolar
21	36	1 st Molar
22	37	2nd Molar
23	38	3rd Molar
24	41	Central Incisor
25	42	Lateral Incisor
26	43	Canine
27	44	1 st Premolar
28	45	2nd Premolar
29	46	1 st Molar
30	47	2nd Molar
31	48	3rd Molar
32	N/A	Bridge
33	N/A	Filling—Crown
34	N/A	Implant

Annotations were performed by an expert under the supervision of a licensed dental professional. Annotator training included dental anatomy and radiograph interpretation to ensure consistency. Certain dental features were intentionally excluded from annotation: root canal treatments and dental braces were not annotated. Fig. 3 illustrates example annotations.

**Fig. 4:**
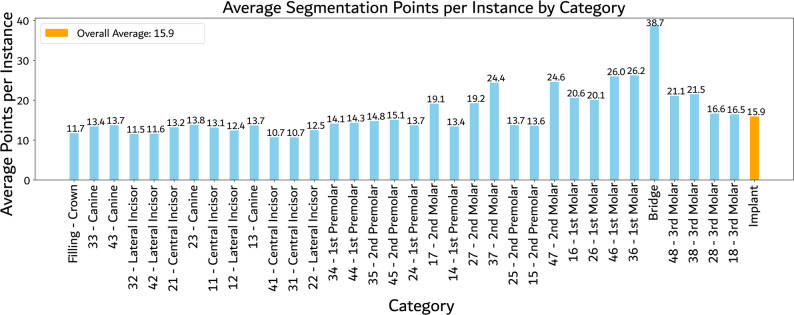
Average segmentation points per instance by category

The average number of polygon points per class was calculated to indicate annotation precision. As shown in Fig. 4, each class exhibits varying levels of detail. Each annotated instance is represented by average 15.9 segmentation points, with the fewest points observed in the 41/31 central incisors and the most in bridges, which average 38.7 points per instance. Annotating the 9,956 instances in the AKUDENTAL dataset required substantial manual labour. Based on an average annotation time of 43.5 seconds per instance, including post annotation control sessions that increased the per-instance effort to 57.4 seconds, the total estimated annotation time amounts to approximately 159 hours.

#### Database structure and annotation formats

The AKUDENTAL dataset is organized to support reproducibility and ease of use across various deep learning frameworks. Provides annotations in multiple formats, including Labelme (.json) for polygon-based segmentation, YOLO (.txt) for object detection tasks, and a COCO-style file (akudental instances.json) containing instance-level segmentation annotations with both bounding boxes and masks.

**Fig. 5:**
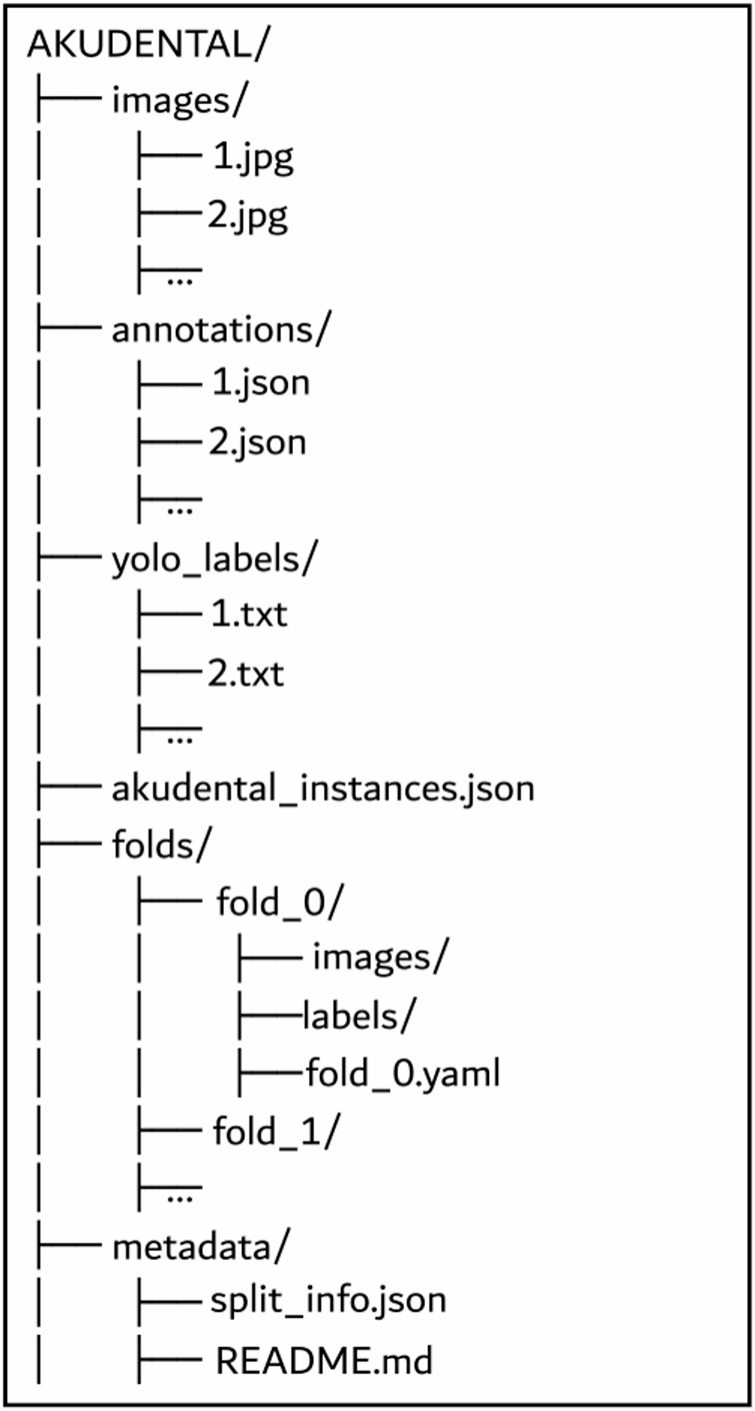
Dataset directory structure overview

The dataset directory structure, as illustrated in Fig. 5, consists of several folders. The folds directory includes five subdirectories (fold 0 through fold 4), each structured for stratified 5-fold cross-validation and containing images and labels folders. The 5-fold stratified cross-validation setup ensures representative sampling, particularly for classes with varying frequencies. The first release of the AKUDENTAL dataset is publicly available for non-commercial academic research via our GitHub repository: www.github.com/melihoz/akudental. The repository contains detailed documentation and scripts for downloading, preprocessing, training, and evaluating models on the dataset.

### Baseline model architectures

To demonstrate the applicability of the AKUDENTAL dataset, we employed several wellknown deep learning models as baselines across object detection, instance segmentation, and semantic segmentation tasks. These models were selected for their established performance in medical and dental imaging domains.

- *Object Detection and Instance Segmentation:*
  - YOLOv11: Modern single-stage architecture designed for efficient real-time object detection and instance segmentation. It has an advanced backbone and head designs that offer improved accuracy and speed over earlier versions [14].
  - Mask R-CNN: A two-stage model that first detects objects and then generates pixel-level masks, enabling precise instance-level analysis of dental structures [15].
- *Semantic Segmentation*:
  - DeepLabV3 + (with ResNet50 backbone): Utilizes atrous spatial pyramid pooling and a decoder module to capture multi-scale context and refine segmentation boundaries [16].
  - UNet-ResNet50: Combines a symmetric encoder-decoder U-Net structure with a ResNet50 encoder to effectively segment fine dental features in panoramic images [17].

### Evaluation metrics

The performance of the models was assessed using task-appropriate metrics across object detection, instance segmentation, and semantic segmentation.

#### Mean Intersection over Union (mIoU)

Mean Intersection over Union (mIoU) shown in Eq. 1 is a standard metric for semantic segmentation. It measures the average overlap between the predicted segmentation masks and the ground truth and is defined as:

1 $${\mathrm{mIoU}}=\frac{1}{N}\sum\limits_{i=1}^{N}\frac{\left|{P}_{i}\cap {G}_{i}\right|}{\left|{P}_{i}\cup {G}_{i}\right|}$$

where *P*_*i*_ and *G*_*i*_ denote the predicted and ground truth masks for class *i*, and *N* is the number of classes.

#### Dice coefficient

The Dice Coefficient, also known as the Sørensen–Dice index Eq. (2), quantifies the similarity between predicted and ground truth segmentation masks:

2 $${\mathrm{Dice}}=\frac{2\left|P\cap G\right|}{\left|P\right|+\left|G\right|}$$

#### Mean Average Precision (mAP)

Mean Average Precision (mAP) is the primary evaluation metric for object detection and instance segmentation. It summarizes the precision-recall curve for multiple intersection- over-union (IoU) thresholds. In our evaluation, mAP is reported at standard thresholds, including IoU@0.50 and IoU@0.75, as well as the averaged mAP over 0.50:0.95.

#### Mean Average Recall (mAR)

Mean Average Recall (mAR) measures the model’s ability to retrieve all relevant objects. It is computed over multiple IoU thresholds and averaged across categories. A higher mAR indicates better sensitivity to object presence and completeness of detection.

#### Precision

Precision refers to the proportion of predicted positive pixels (or objects) that are correctly identified Eq. (3):

3 $$\mathrm{Precision}=\frac{\mathrm{TP}}{\mathrm{TP}+\mathrm{FP}}$$

#### Recall

Recall measures the proportion of actual positive pixels (or objects) correctly predicted Eq. (4):

4 $$Recall= \frac{\mathrm{TP}}{{\mathrm{TP}}+{\mathrm{FN}}}$$

#### Accuracy

Accuracy is the proportion of correctly classified pixels across the entire image Eq. (5):

5 $$Accuracy=\frac{{\mathrm{TP}}+{\mathrm{TN}}}{{\mathrm{TP}}+{\mathrm{TN}}+{\mathrm{FP}}+{\mathrm{FN}}}$$

All metrics were averaged across the validation and test sets to report overall performance.

### Data augmentation

To improve generalization and reduce overfitting, several data augmentation techniques were applied during training:

- Random rotations up to 5 degrees (both clockwise and counterclockwise)
- Mild Gaussian blurring to simulate slight imaging imperfections
- Random cropping and scaling to increase robustness to size and position variance
- Normalization to ensure consistent intensity distribution across samples

These augmentations were only applied to training data. Validation and test sets were processed using normalization and resizing alone, ensuring a consistent and unbiased evaluation protocol.

## Experiments

### Experimental setup

Experiments were performed using PyTorch and relevant model implementations: YOLOv11 via Ultralytics, Mask R-CNN via Detectron2, and segmentation models via Torchvision. Input images were resized to 512 × 512 pixels and normalized using ImageNet statistics. Mixed- precision training was utilized for efficiency.

Performance was assessed using a 5-fold cross-validation protocol. For each fold, 80% of the images were allocated to the training set. The remaining 20% of images were held out and then evenly divided to create separate validation and test subsets, each constituting 10% of the total dataset. This procedure was repeated five times, ensuring that each image served in a validation set once and, in a test, set once across the folds.

#### Training protocol

All models were trained for 150 epochs using the Adam optimizer (initial learning rate: 10^*−*3^, weight decay: 10^*−*5^). The ReduceLROnPlateau scheduler was employed (patience=10 epochs, factor=0.1). Batch sizes were set to 4 for segmentation models (UNet, DeepLabV3+) and 4 for detection and instance segmentation models (YOLO, Mask R-CNN).

Loss functions were task-specific: cross-entropy loss for semantic segmentation, binary cross-entropy with mask predictions for instance segmentation, and classification plus bounding-box regression losses for object detection tasks were used.

#### Statistical analysis

Performance differences between models were assessed using paired t-tests conducted at the per-image level for segmentation metrics (Dice, mIoU) and object-level for detection metrics (mAP). Statistical significance was defined at *α* = 0*.*05. Results reported are averages ± standard deviations across all five folds.

### Evaluation overview

Four models: YOLO, Mask R-CNN, UNet-ResNet50, and DeepLabV3+ were evaluated across three distinct tasks: object detection, instance segmentation, and semantic segmentation. Metrics were averaged across folds.

#### Semantic segmentation results

Semantic segmentation was evaluated using DeepLabV3+ and UNet architectures, both initialized with ImageNet pretrained weights. Quantitative results across 5-fold cross-validation are summarized in Table 3 Statistical analysis revealed no statistically significant differences between DeepLabV3+ and UNet in terms of mIoU (validation *p* = 0*.*444, test *p* = 0*.*569) and Dice coefficient (validation *p* = 0*.*526, test *p* = 0*.*616). As indicated in the table, UNet displayed slightly higher average metrics.

**Table 3:** Performance metrics of DeepLab and UNet models across 5 folds on validation and test sets

Model	Fold	mIoU		Dice		Precision		Recall		Accuracy	
		Val	Test	Val	Test	Val	Test	Val	Test	Val	Test
DeepLab	1	0.6009	0.669	0.6873	0.7557	0.7458	0.7856	0.6735	0.7475	0.9708	0.9731
	2	0.6727	0.6589	0.7609	0.7458	0.7945	0.7872	0.752	0.7305	0.9722	0.9723
	3	0.6916	0.6401	0.7774	0.7243	0.8027	0.7565	0.7752	0.7202	0.9743	0.971
	4	0.6286	0.6709	0.7113	0.7572	0.7565	0.7985	0.695	0.7453	0.97	0.9702
	5	0.6761	0.6246	0.7611	0.7092	0.7841	0.7468	0.7622	0.699	0.9731	0.9712
	Avg	0.654	0.6527	0.7396	0.7384	0.7767	0.7749	0.7316	0.7285	0.9721	0.9716
UNet	1	0.6099	0.6655	0.6954	0.75	0.745	0.7848	0.6807	0.7387	0.9717	0.9731
	2	0.6922	0.6749	0.7775	0.7618	0.8008	0.7986	0.7724	0.746	0.9745	0.9738
	3	0.6931	0.6406	0.7757	0.7255	0.8008	0.7612	0.7703	0.7167	0.9754	0.9717
	4	0.649	0.6782	0.7304	0.7619	0.7638	0.7999	0.718	0.7505	0.9724	0.9719
	5	0.6873	0.6484	0.7712	0.7323	0.8039	0.7774	0.7604	0.714	0.9739	0.9736
	Avg	0.6663	0.6615	0.75	0.7463	0.7829	0.7844	0.7404	0.7332	0.9736	0.9728

### Object detection

**Fig. 6:**
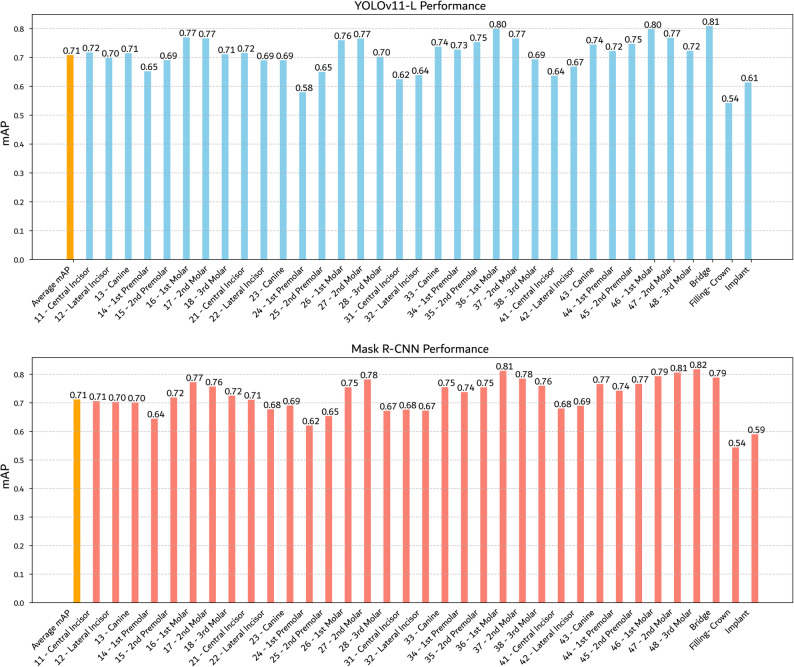
mAP comparison per class: Mask R-CNN vs. YOLOv11-L

We evaluated several object detection architectures, including YOLOv11 (Small, Medium, and Large) and Mask R-CNN, on the AKUDENTAL dataset. All models were trained under identical conditions and evaluated using COCO-style metrics (mAP, mAP@50, and mAP@75), averaged over five cross-validation folds. As shown in Table 4, performance differences among the YOLO variants were minimal, with only numerically small gains for larger models. A paired t-test comparing YOLOv11-Large and Mask R-CNN demonstrated no statistically significant differences in detection performance. The analysis yielded *p*-values on the validation set of *p* = 0.541 (mAP), *p* = 0.832 (mAP@50), and *p* =0.173 (mAP@75), and on the test set of *p* = 0.747 (mAP), *p* = 0.389 (mAP@50), and *p* = 0.665 (mAP@75). Class-wise performance comparisons are provided in Fig. 6.

**Table 4:** Average validation and test performance of all object detection models

Model	mAP			mAP@50			mAP@75	
	Val	Test		Val	Test		Val	Test
YOLOv11 Small	0.71	0.69		0.94	0.93		0.85	0.84
YOLOv11 Medium	0.72	0.70		0.95	0.94		0.87	0.84
YOLOv11 Large	0.72	0.71		0.95	0.94		0.87	0.85
Mask R-CNN	0.72	0.71		0.95	0.95		0.85	0.84

### Instance segmentation

**Fig. 7:**
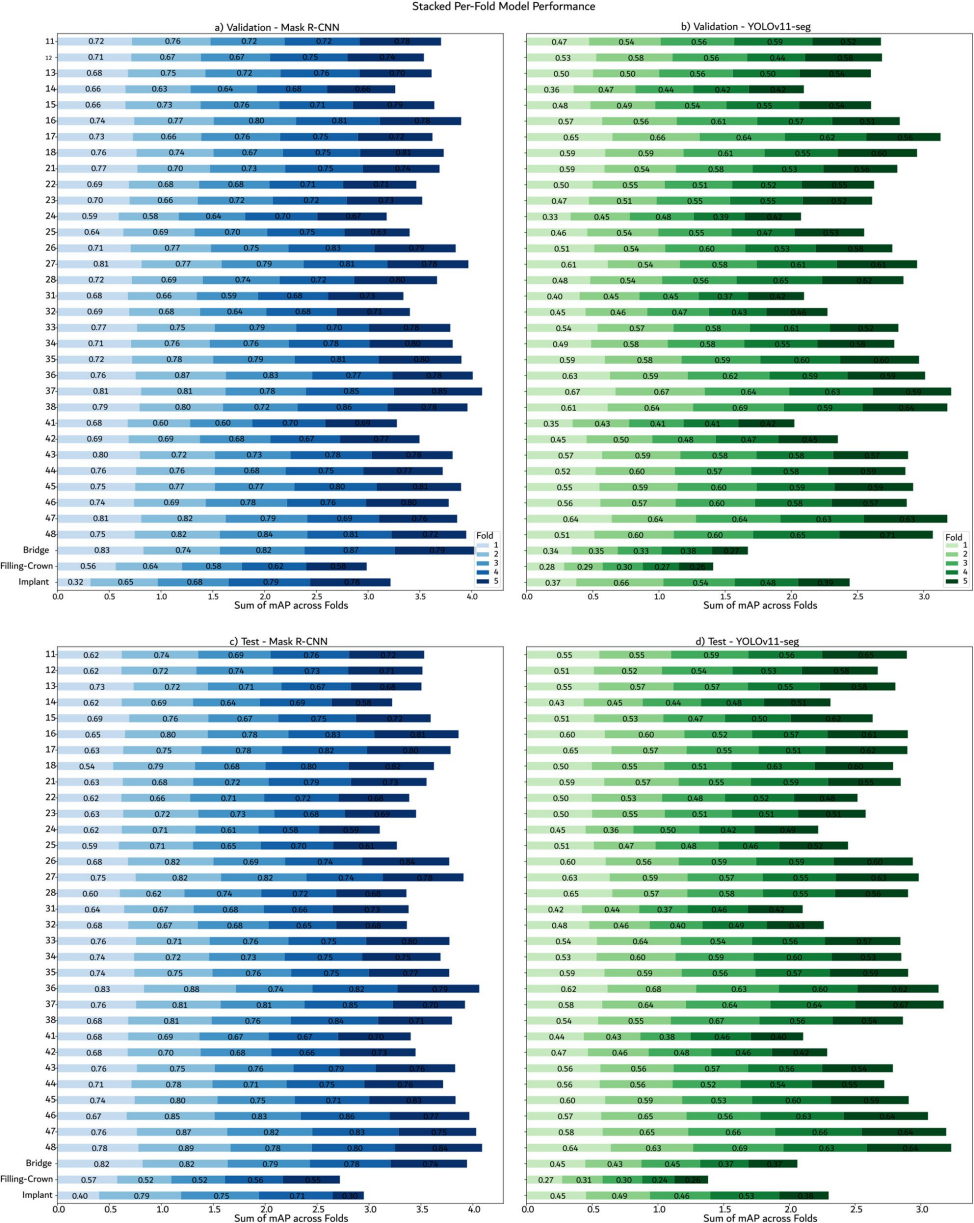
Comparison of per-fold mAP scores for Mask R-CNN and YOLOv11-seg. The plots show stacked bar charts for **a**-**b** validation and **c**-**d** test datasets, with each segment representing the performance of an individual fold

Instance segmentation experiments were conducted using Mask R-CNN and the YOLOv11 instance segmentation model. The results, presented in Table 5, include a bounding box mAP of 0.72 (validation) and 0.71 (test), and a segmentation mAP of 0.67 (validation and test). For the instance segmentation task, a statistical comparison between our YOLOv11-seg model and the baseline Mask R-CNN was conducted using a paired *t*-test on the 5-fold cross-validation results. This analysis was performed independently for both the validation and test datasets, with the detailed metrics presented in Table 6. The results for each class in each fold is reported in Fig. 7.

**Table 5:** Average object detection and segmentation performance for Mask R-CNN and YoloV11-seg models

Model	Split	mAP	mAP@50	mAP@75	mAP Segm	mAP@50 Segm	mAP@75 Segm
Mask R-CNN	Validation	0.72	0.95	0.85	0.67	0.94	0.84
Mask R-CNN	Test	0.71	0.95	0.84	0.67	0.94	0.83
YOLOv11-seg	Validation	0.68	0.94	0.81	0.55	0.91	0.59
YOLOv11-seg	Test	0.68	0.94	0.81	0.55	0.91	0.59

**Table 6:** Cross-dataset detection performance (YOLOv11-L trained on AKUDENTAL, evaluated on external datasets)

Evaluation Dataset	Configuration	mAP	mAP@50	mAP@75
_Tufts_	Multiclass	0.42	0.83	0.35
	Binary (Excl. Bridges/Crowns/Fillings)	0.54	0.92	0.57
_DENTEX_	Multiclass	0.34	0.86	0.17
	Binary (Excl. Bridges/Crowns/Fillings)	0.37	0.93	0.20
Dual-labeled	Multiclass	0.71	0.94	0.86
	Binary (Excl. Bridges/Crowns/Fillings)	0.72	0.97	0.87

The findings were highly consistent across both splits. Mask R-CNN demonstrated a statistically significant improvement in overall segmentation mAP (validation *p* = 0.00062, test *p* = 0.00003) and at the stricter mAP@75 threshold (validation *p* = 0.00018620, test *p* = 0.00033). In contrast, no statistically significant difference was observed at the more lenient mAP@50 threshold, with *p*-values of *p* = 0.168 for the validation set and *p* = 0.086 for the test set.

These results suggest that while both models are equally proficient at generating a coarse segmentation that correctly localizes instances, the Mask R-CNN architecture provides substantially greater precision in delineating the exact object boundaries.

### Cross-dataset evaluation and domain shift analysis

To assess the representational capacity and robustness of the AKUDENTAL dataset under external domain shifts, we conducted cross-dataset experiments using three publicly available dental panoramic radiograph datasets: the Tufts Dental Radiograph dataset, the DENTEX dataset, and the Dual-labeled dataset. Each external dataset presented unique domain shifts, primarily due to differences in annotation protocols, class definitions, and included dental conditions.

The Tufts dataset consists of 1,000 panoramic images annotated with 52 classes, including primary teeth and implants, with implants labeled according to the replaced teeth rather than as a distinct class. The DENTEX dataset includes 634 panoramic radiographs with bounding box annotations for 32 tooth numbering classes. The Dual-labeled dataset is the largest, containing 2066 images with annotations for instance segmentation including 32 tooth numbering classes are used [4]. State labels are not included for the datasets.

Given these annotation disparities, we defined two experimental configurations to evaluate the cross-dataset experiment thoroughly:

- *Multiclass Configuration:* All annotated tooth types were kept. Dental procedures (fillings, crowns, and bridges) were excluded from the AKUDENTAL training data. Implants are included for the Tufts excluded for the rest.
- *Binary Configuration:* A simplified setup where all remaining teeth and implant annotations were merged into a single, unified class. This approach minimizes annotation differences, providing a clearer evaluation of general tooth detection performance.

YOLOv11-L was selected as the representative baseline model, based on its performance detailed in Table 4.

Table 6 presents the detection performance of YOLOv11-L trained on AKUDENTAL and directly evaluated on the Tufts, DENTEX, and Dual-labeled datasets without any retraining or fine-tuning.

AKUDENTAL and directly evaluated on Tufts and DENTEX datasets without retraining or fine-tuning. While evaluating the Dual-labeled dataset, the model achieved a multiclass mAP of 0.71. On the Tufts dataset, YOLOv11-L achieved a mAP of 0.42 under the multiclass configuration. Performance in the binary setup resulted in a mAP of 0.54. When evaluated on the DENTEX dataset, YOLOv11-L showed similar trends. Multiclass evaluation yielded a mAP of 0.34, while binary evaluation resulted in an mAP of 0.37.

**Fig. 8:**
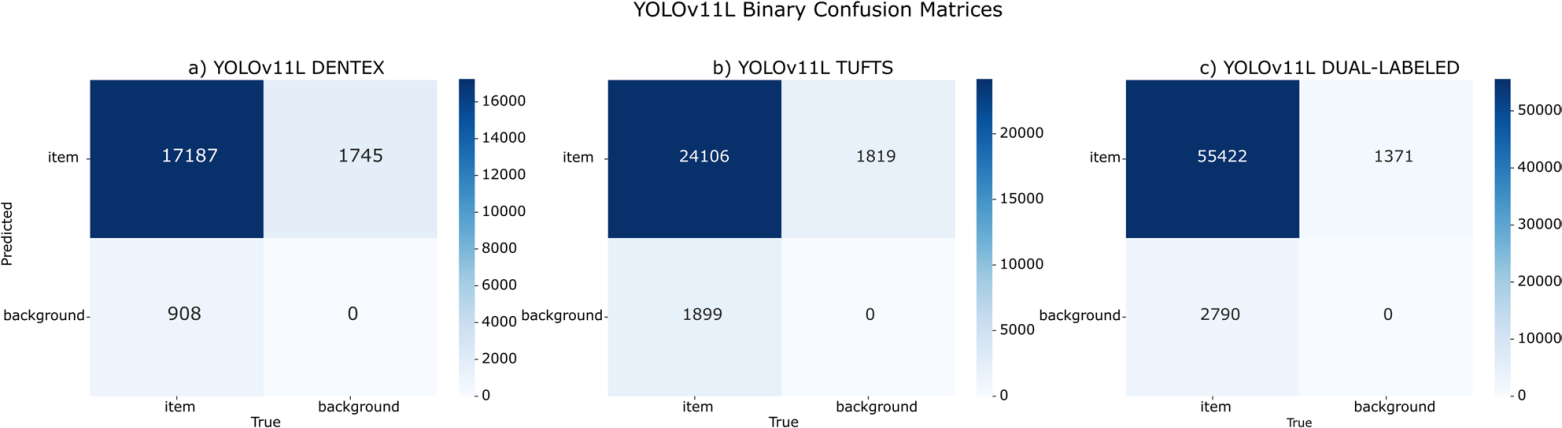
**a** DENTEX Dataset Binary Confusion Matrix, **b** Tufts Dataset Binary Confusion Matrix, **c** Dual-labeled Dataset Binary Confusion Matrix

Figure 8 further elucidates these outcomes, depicting confusion matrices under the binary scenario for the Tufts and DENTEX evaluations. It reveals consistent misclassifications attributable to differences in annotation protocols and domain variability, emphasizing specific domain-adaptation challenges in dental radiograph analysis.

## Discussion

The experimental results offer insights into model performance and dataset features within dental panoramic X-ray image analysis. This section synthesizes quantitative results and qualitative observations from evaluations on the AKUDENTAL dataset and externally on the Tufts, DENTEX, and Dual-labeled datasets, explicitly addressing domain generalization challenges and methodological implications.

### Model performance on AKUDENTAL

On the AKUDENTAL dataset, evaluated models consistently demonstrated strong performance across object detection, instance segmentation, and semantic segmentation tasks. For object detection, Mask R-CNN and YOLOv11 models yielded comparable overall mAP values, suggesting Mask R-CNN’s two-stage architecture offers no specific advantages for bounding box localization in this context.

Semantic segmentation experiments displayed no statistically substantial differences between UNet and DeepLabV3 + (mIoU: validation *p* = 0.444, test *p* = 0.569; Dice: validation *p* = 0.526, test *p* = 0.616). Nevertheless, UNet demonstrated modestly better numerical performance, likely due to its effective encoder-decoder structure with skip connections preserving fine structural details. Convergence characteristics also suggested UNet offers smoother convergence and improved stability on this specific domain.

Instance segmentation experiments were conducted using Mask R-CNN, a two-stage detection and segmentation model, and the YOLOv11-seg, a single-stage instance segmentation model. The results, summarized in Table 5, show bounding box mAP values of 0.72 (validation) and 0.71 (test), and segmentation mAP values of 0.67 for both splits using Mask R-CNN. To rigorously compare the models, we performed a paired t-test on the 5-fold cross-validation results, analyzed independently for both validation and test datasets (detailed in Table 6).

The analysis revealed that Mask R-CNN significantly outperforms YOLOv11-seg in overall segmentation mAP (validation *p* = 0.00062, test *p* = 0.00003) and at the stricter mAP@75 threshold (validation *p* = 0.00018620, test *p* = 0.00033). Conversely, no significant difference was observed at the more lenient mAP@50 threshold (*p* = 0.168 for validation, *p* = 0.086 for test).

These findings suggest that while both models are equally effective at generating coarse segmentations that correctly localize dental instances, the two-stage architecture of Mask R-CNN provides substantially greater precision in delineating exact object boundaries compared to the single-stage YOLOv11-seg. This difference is important for where detailed boundary accuracy is critical.

**Fig. 9:**
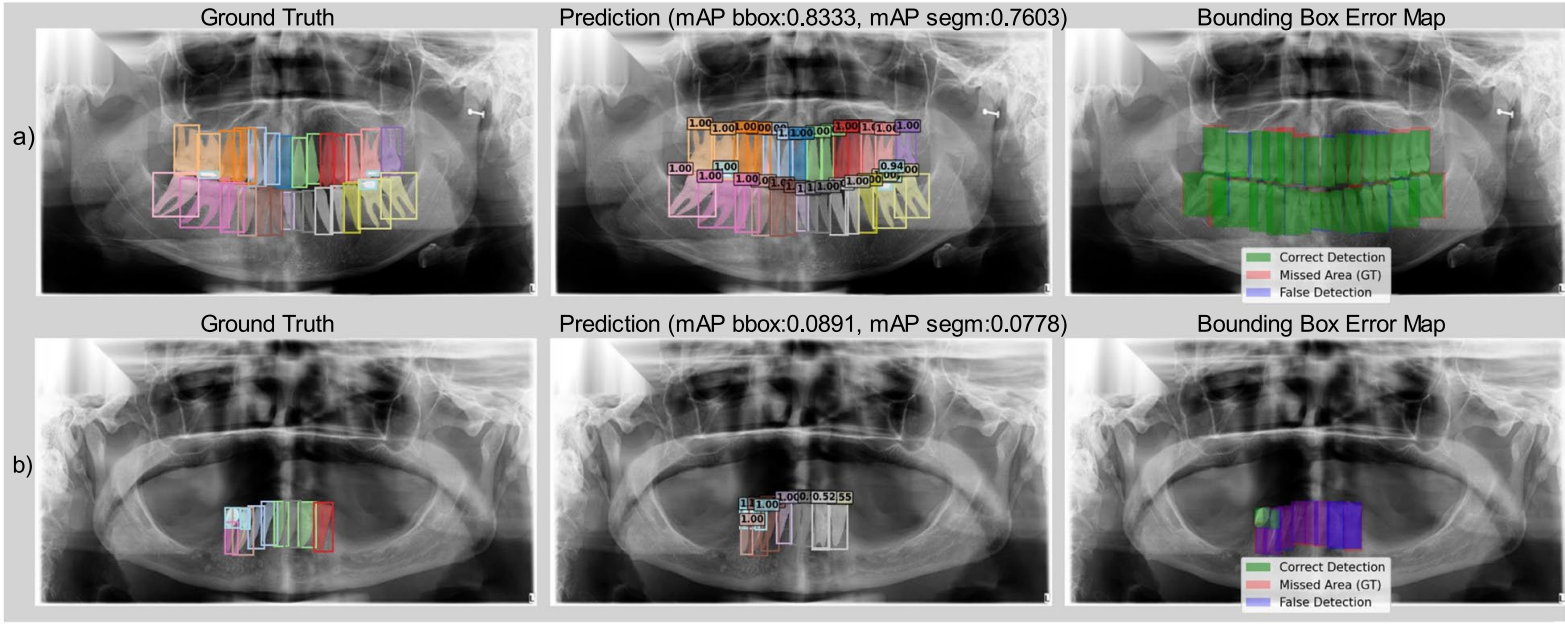
Qualitative examples of model performance on AKUDENTAL **a** successful prediction in a dentition with most teeth present; **b** challenges in tooth numbering when extensive tooth loss is evident

Qualitative analysis, as exemplified in Fig. 9a, shows that when most teeth are present, models trained on AKUDENTAL generally predict tooth numbers and locations very effectively. However, as seen in Fig. 9b, performance in tooth numbering can degrade significantly in cases with extensive tooth loss, where contextual cues for numbering are diminished.

### Cross-dataset generalization, domain shift, and annotation discrepancies

Cross-dataset evaluation, as summarized in Table 5, emphasized significant domain adaptation challenges stemming from differences in annotation conventions and dataset characteristics. When AKUDENTAL-trained models were directly tested on the Tufts and DENTEX datasets without class-tuning, noticeable performance declines were observed, particularly under multiclass configurations. For instance, YOLOv11-L achieved mAP scores of 0.42 (Tufts) and 0.34 (DENTEX) in multiclass setups. In contrast, the evaluation on the Dual-labeled dataset yielded a substantially higher multiclass mAP of 0.71.

**Fig. 10:**
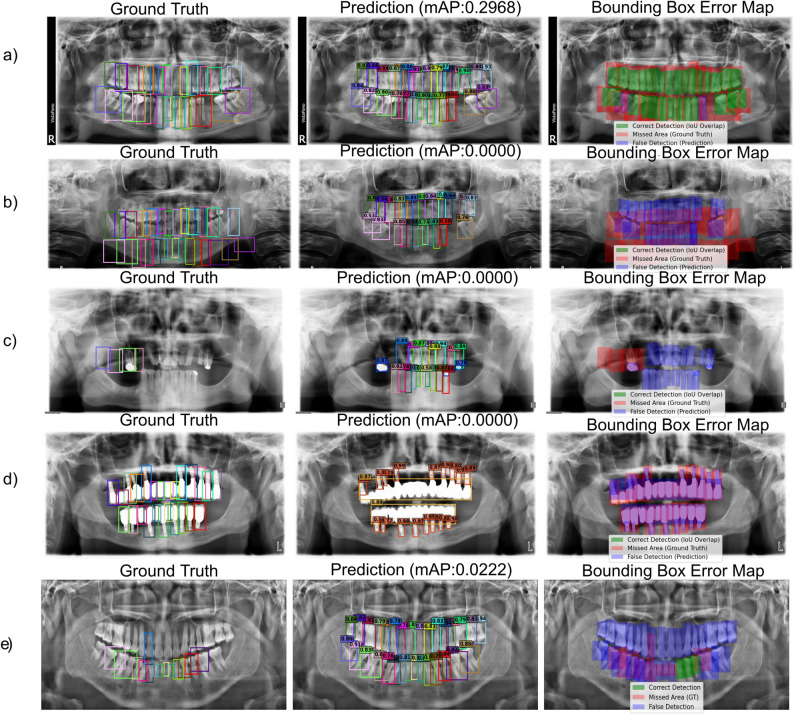
Illustrative challenges in cross-dataset evaluation: **a** Impact of loose DENTEX bounding boxes on mAP despite correct predictions. **b** Incorrect/missing ground truth in DENTEX. **c** Incorrect/missing ground truth in Tufts. **d** Correct predictions by AKUDENTAL-trained model on a Tufts image with implants/bridges yield zero mAP due to differing class definitions. **e** Incorrect/missing ground truth in Dual-labeled

This performance gap can be attributed primarily to annotation style and class definition alignment. The Dual-labeled dataset adopts a 32-tooth multiclass scheme with tight bounding boxes that closely mirror AKUDENTAL, resulting in better transferability. In contrast, the DENTEX dataset, though also 32-class, uses more relaxed bounding boxes around teeth, leading to penalization of visually correct predictions that fall partially outside ground-truth regions (Fig. 10a). The Tufts dataset further diverges by not only including primary teeth as separate classes but also treating implants as regular teeth depending on their location. These additional categories, absent in AKUDENTAL, introduce class mismatches and inflate the false positive rate when evaluated cross-dataset (Fig. 10d).

The quantitative evidence for these annotation effects is visible in the large discrepancies between mAP@50 and mAP@75. In Tufts (0.83 vs. 0.35) and DENTEX (0.86 vs. 0.17), performance collapses when moving from IoU=0.5 to IoU=0.75, indicating loose or inconsistent annotation borders. In contrast, the Dual-labeled dataset maintains consistently high scores (0.94 vs. 0.86), confirming the role of tighter and more standardized boundary annotations.

When the annotation complexity was reduced to binary configurations (tooth vs. background, excluding specific procedures), cross-domain generalization improved across all datasets, with mAP on Tufts increasing to 0.54, on DENTEX to 0.37, and on the Dual-labeled dataset to 0.72. The marginal improvement on the Dual-labeled dataset from multiclass (0.71) to binary (0.72) suggests that inter-class confusion was not the limiting factor. Instead, annotation boundary precision and taxonomy alignment dominated performance differences.

Cross-dataset evaluation revealed that the observed limitations arose from multiple factors rather than model capacity alone. Current results indicate that domain shift remains a central issue, with loose bounding boxes in DENTEX often reducing mAP despite correct predictions and taxonomic differences such as Tufts treating implants as natural teeth leading to penalization even when detections were accurate (Fig. 10d). While occasional missing or false annotations were also observed in all datasets (Fig. 10b-e), these cases were relatively rare and should be seen as secondary contributors rather than the primary source of performance discrepancies.

### AKUDENTAL dataset limitations

While AKUDENTAL provides detailed and expert annotations, the dataset size remains limited with 333 panoramic images, which may constrain the generalizability and robustness of trained models, particularly for rare or complex dental conditions. The relatively small sample size may limit the statistical power of experiments and the diversity of anatomical and pathological variations represented. This is a common challenge in medical imaging datasets due to the difficulty of expert annotation and data collection.

Annotations were performed by a single expert annotator under the supervision of a licensed dental professional. Although the annotator underwent structured training in dental anatomy and radiographic interpretation to ensure consistency, the use of a single annotator precludes inter-annotator agreement (IAA) analysis. We acknowledge this as a methodological limitation that may affect the assessment of annotation reliability.

Additionally, occasional ambiguities in annotated boundaries may arise in complex dental regions with overlapping structures or subtle restorations. The dataset currently does not cover all clinically relevant dental findings, such as root canal fillings, periodontal artifacts, or a wide spectrum of pathological conditions. These limitations should be considered when applying models trained on AKUDENTAL for broader diagnostic systems.

### Future directions

Future extensions of AKUDENTAL should emphasize dataset expansion by incorporating a broader spectrum of dental conditions, procedural annotations, and demographic diversity.

Specifically, to address the observed degradation in tooth numbering performance in scenarios with extensive tooth loss (as highlighted by Fig. 9b), including more examples of such cases will be a priority to improve model robustness in these challenging situations. Enhancing annotation precision, particularly in ambiguous regions and complex restorative cases, would also substantially improve training quality and model reliability. Exploring semi-supervised or self-supervised learning techniques leveraging the existing labeled data alongside a larger pool of unlabeled images could also be a valuable direction for improving model generalization and reducing annotation burden. Establishing clearer, more universally adopted annotation standards across the dental imaging community would be pivotal for advancing the field and enabling more robust and comparable research. This version of the dataset (v1.0) serves as an initial release. Future versions of AKUDENTAL will incorporate annotations from multiple expert annotators and include inter-annotator agreement (IAA) analysis, aiming to further improve the dataset’s quality, transparency, and reliability for the research community.

## Data Availability

The AKUDENTAL dataset, along with all code for data processing, training, and evaluation, is publicly available for non-commercial academic research at: https://www.github.com/melihoz/akudental. The repository provides detailed instructions for accessing and using the data and materials associated with this study.

## Abbreviations

AI: Artificial Intelligence
CNNs: Convolutional Neural Networks
CT: Computed Tomography
FDI: Fédération Dentaire Internationale
mAP: Mean Average Precision
mAR: Mean Average Recall
mIoU: Mean Intersection over Union
RCT: Root Canal Treatment
TP: True Positive
FP: False Positive
FN: False Negative
